# Impaired fatty acid metabolism in AKI: experimental evidence – a narrative review

**DOI:** 10.1186/s12882-026-05166-8

**Published:** 2026-07-13

**Authors:** Johanna Gruzla, Stefan Erfurt, Benedikt Marahrens, Karsten-Henrich Weylandt, Oliver Ritter, Daniel Patschan

**Affiliations:** 1 Department of Internal Medicine I - Cardiology, Nephrology and Internal Intensive Care Medicine, University Hospital Brandenburg, Brandenburg Medical School (Theodor Fontane), Hochstraße 29, 14770 Brandenburg an der Havel, Germany; 2 Division of Medicine, Department of Gastroenterology, Metabolism and Oncology, University Hospital Ruppin-Brandenburg, Brandenburg Medical School, Neuruppin, Germany; 3 Faculty of Health Sciences (FGW), Joint faculty of the University of Potsdam, the Brandenburg Medical School Theodor Fontane and the Brandenburg Technical University Cottbus-Senftenberg, Cottbus, Germany

**Keywords:** AKI, Fatty acids, FA, Oxidation, Endogenous, Exogenous

## Abstract

**Background:**

Acute kidney injury (AKI) is a common and serious complication of hospitalized patients worldwide. In most cases, the syndrome is characterized by dysfunction/structural damage of the tubular epithelial cells. The metabolic feature is a reduction in cellular adenosine triphosphate (ATP) synthesis as a result of decreased fatty acid oxidation. Stabilization of the fatty acid (FA) oxidation process may influence the course of AKI, whether caused by endogenous or exogenous factors. In this narrative review, we discuss experimental evidence on the modulation of fatty acid metabolism in AKI.

**Methods:**

Narrative review article. The following databases were searched for references: PubMed, Web of Science, Cochrane Library, Scopus. The period lasted from 2010 until May 2026.

**Results:**

Finally, 16 articles on the modulation of fatty acid metabolism in experimental AKI were identified. Disturbances in the FA oxidation process are associated with an aggravation of renal dysfunction in AKI, and lipids accumulate in the renal tissue itself. The consequences of exogenous fatty acid intake depend on the structural characteristics of the metabolites applied, as well as on the pattern of renal damage and the relevant pathophysiological processes.

**Conclusions:**

Under certain conditions, adequate fatty acid oxidation may be considered nephroprotective; however, the complexity of the syndrome’s pathophysiology must always be taken into account. Distinct fatty acids have the potential to offer therapeutic benefits in AKI cases. However, the efficacy of an FA-based therapeutic approach depends on two main factors: the chemical structure of the substances used and the nature of the underlying kidney disease.

## Introduction

Acute kidney injury (AKI) is a prevalent problem among hospitalized patients worldwide. The incidence of AKI varies geographically, exhibiting regional differences of 5% to 30% [1]. Individuals receiving treatment in intensive care units are afflicted with an acute disorder of excretory kidney function in up to 50% of cases, and sometimes, even more frequently [2]. Moreover, the mortality rate associated with this syndrome has remained relatively constant over the past three decades.

The etiology of AKI exhibits significant heterogeneity; the most prevalent cause in absolute terms is a critical, albeit usually only temporary, reduction in renal blood flow [3]. This condition often leads to substantial functional deterioration and, in some cases, structural damage to the tubular epithelium. This condition, formally known as acute tubular necrosis (ATN), has also been observed to manifest in conjunction with certain pharmaceutical treatments. It should be noted, however, that the term “reduction in renal blood flow” certainly oversimplifies the complexity of the in vivo processes. It would likely be more accurate to speak of a perfusion imbalance or disturbance. In heart failure, venous congestion (with arterial blood flow remaining intact) can also induce or exacerbate AKI. It is not without reason that, in the context of cardiac compensation therapy, excretory renal function stabilizes in many cases [4, 5]. The concept of perfusion imbalance is even more justified in the case of septic AKI. This can manifest as early as the stage of generalized hyperperfusion and is then based on regional imbalances in microcirculatory flow or is also a consequence of regional inflammation [6]. Both processes, microvasculopathy and inflammation, are sustained by endothelial dysfunction [7]. Proximal tubular epithelial cells are highly vulnerable to ischemia because of their large active reabsorption workload and their location in the outer medulla [8]. Early ischemic changes include cellular swelling, detachment from the basement membrane, tubular casts and debris that can obstruct lumens, interstitial edema, tubular dilation, and, in severe cases, acute tubular necrosis (ATN) [9]. Ischemia damages mitochondria, causing ATP depletion, increased anaerobic glycolysis and lactate production, and loss of ion transport and concentrating ability; irreversible mitochondrial injury marks the “point of no return” when persistent ATP loss leads to membrane failure and cell necrosis [10]. Restoring perfusion by rehydration can reverse injury if done before this point, but reperfusion can also provoke a pro-inflammatory response that worsens damage. Successful recovery during reperfusion depends mainly on effective circulating volume.

In particular, within the intensive care setting, AKI frequently arises from a combination of reduced perfusion, the impact of nephrotoxic medications, and the detrimental intrarenal and systemic consequences of sepsis. The pathogenesis of ischemic, toxic, or combined ATN is intricate and involves multiple biological processes [11]. However, disturbances of lipid and especially fatty acid homeostasis are particularly salient for the initiation and maintenance of the syndrome. Possible disruptions include impaired fatty acid oxidation (FAO), alterations in lipid droplet formation, and changes in membrane phospholipids, particularly sphingolipids [12]. In addition, utilizing lipid metabolism products as biomarkers may facilitate early detection of AKI and allow for assessment of its severity, emphasizing the importance of correcting lipid metabolism abnormalities to improve patient prognosis [12].

The subject of fatty acid metabolic disturbances in acute kidney injury alone is extensive. It encompasses the potential pathogenic implications of these disorders in AKI, as well as the modulation of pathological, AKI-associated processes by targeting the FA metabolism through exogenous measures. Finally, it includes the quantification of fatty acids and their metabolites for diagnostic purposes. A thorough examination of these three subjects is beyond the scope of this article. Therefore, mainly experimental data will be recapitulated with the aim to identify pathognomic patterns of endogenous fatty acid metabolic disorders in AKI and to discuss possible modulations of fatty acid metabolism by exogenous measures. Since the two etiological determinants - ‘hypoperfusion’ or ‘ischemia’ as well as ‘nephrotoxicity’ or ‘tubular toxicity’ - are of dominant relevance in everyday clinical practice, the references were also selected according to these aspects.

## Methods

**Fig. 1:**
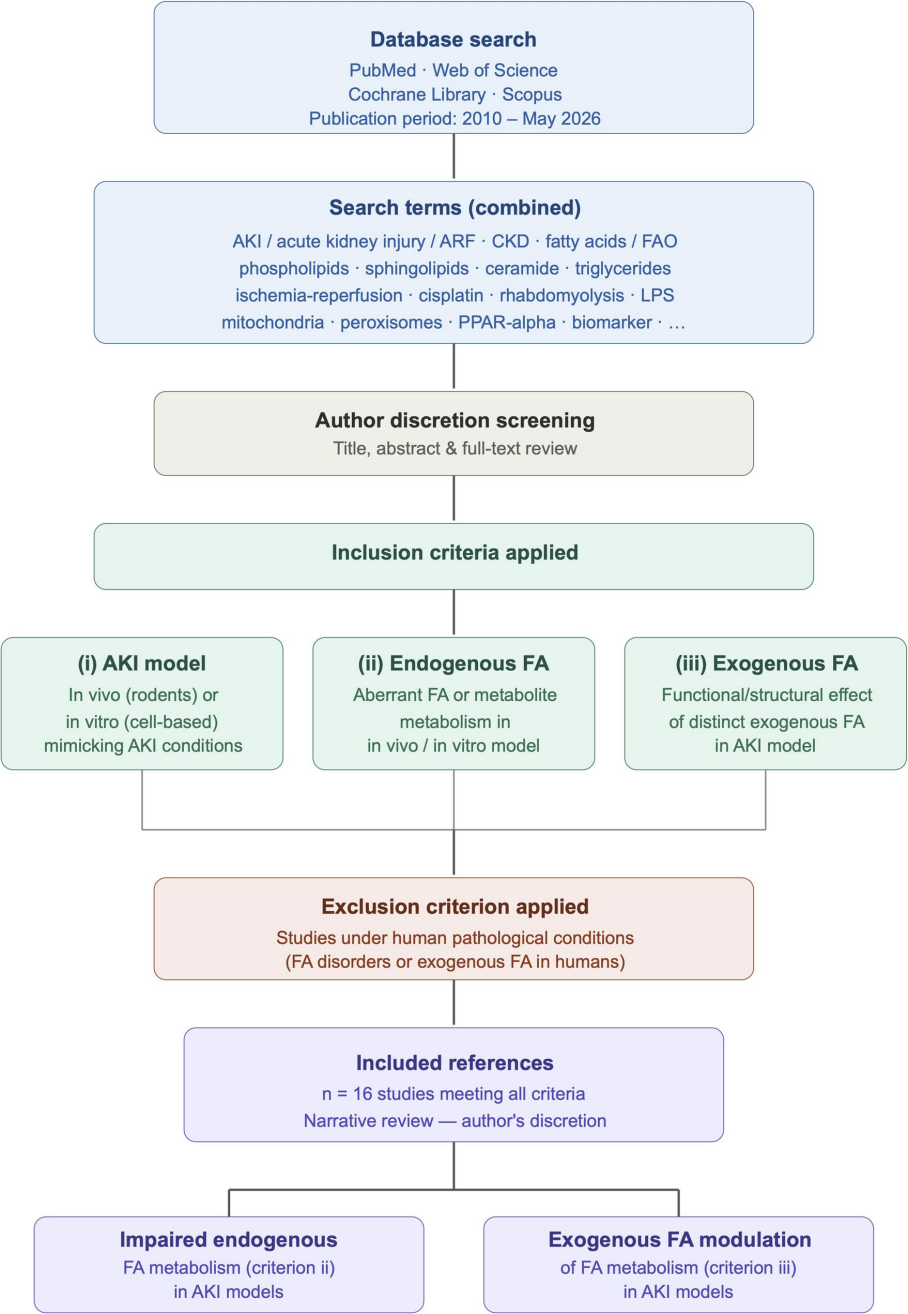
Search strategy

It is a narrative review article. All references were chosen at the author´s discretion. The following databases were searched for references: PubMed, Web of Science, Cochrane Library, Scopus. The search encompassed relevant literature published within the period from 2010 until May 2026. The following search terms were used in different combinations: ‘acute kidney injury’, ‘AKI’, ‘acute renal failure’, ‘ARF’, ‘chronic kidney disease’, ‘CKD’, ‘fatty acids’, ‘FA’, ‘fatty acid oxidation’, ‘FAO’, ‘phospholipids’, ‘sphingolipids’, ‘ceramide’, ‘lipids’, ‘triglycerides’, ‘biomarker’, ‘exogenous’, ‘endogenous’, ‘tubulopathy’, ‘proximal renal tubule’, ‘PRT’, ‘ischemia’, ‘ischemia-reperfusion’, ‘ischemia-reperfusion injury’, ‘hypoperfusion’, ‘nephrotoxic’, ‘tubulotoxic’, ‘LPS’, ‘cisplatin’, ‘aristocholic acid’, ‘rhabdomyolysis’, ‘microvasculopathy’, ‘inflammation’, ‘interstitial inflammation’, ‘mitochondria’, ‘peroxisomes’, ‘perixisomal’, ‘ATP’, ‘ATP depletion’, ' peroxisome proliferator-activated receptor’, ‘PPAR’, ‘PPAR-alpha’. Eligibility criteria for study inclusion were: (i) an experimental AKI study - conducted either in vivo using rodents (such as mice or rats) or in vitro using cell-based models - that mimics AKI-associated microenvironmental conditions; (ii) an analysis of aberrations in the metabolism of one or more distinct fatty acids or their metabolites within an in vivo or in vitro model of injury (section ‘impairment of endogenous fatty acid metabolism’); (iii) an analysis of the functional or structural consequences of using distinct exogenous fatty acids or their metabolites in an in vivo or in vitro model of AKI (section ‘exogenous modulation of fatty acid metabolism’). Exclusion criteria were: any analysis of fatty acid metabolic disorders or the effects of administering distinct exogenous fatty acids or their metabolites under human pathological conditions. Ultimately, a total of *n* = 16 references were identified that met the criteria. A literature search based on the Prisma criteria [13] was not conducted, as this article is a narrative review. Figure 1 illustrates the search process graphically.

### Acute kidney injury

#### Definition and outcomes

The KDIGO guideline on acute kidney injury was published over 12 years ago [14]. Consequently, the diagnostic criteria delineated in the guideline remain the prevailing standard for defining the syndrome. The primary emphasis is on acute increases in serum creatinine concentration, whether occurring over a 48-hour period (criterion 1) or over the course of seven days (criterion 3). Criterion 3 is distinguished by a substantial reduction in urine production, and the fulfillment of one criterion is sufficient. It is highly probable that subsequent criteria will encompass the cumulative evaluation of novel biomarkers [15–17]. Serum creatinine is expected to retain its role as an indicator of the glomerular filtration rate, while markers of structural damage will most likely be considered from the extensive group of biomarkers that have been identified to date. Acute kidney injury (AKI) frequently complicates the conditions of hospitalized patients, with an incidence ranging from 5 to 30% (1). Intensive care unit patients are disproportionately affected, with rates exceeding 50% (2). In this group, the acute mortality rate is also > 50% with an exceptionally high mortality risk (up to 100% [18]) in certain situations. In addition to the acute deterioration in prognosis, the long-term outcome is reduced. Individual episodes of AKI have been demonstrated to increase the risk of death over the years following the acute event [19]. Moreover, patients with AKI are more prone to developing chronic kidney disease, and the probability of this development increases with the severity of AKI [20].

### The pathogenetic role of fatty acid metabolism in AKI

At this juncture, it is not possible to provide a satisfactory recapitulation of the pathogenesis of ischemic, toxic, or combined ATN. However, it is crucial to acknowledge the primacy of three major pathogenetic processes: tubulopathy, intrarenal microvasculopathy, and the secondary inflammatory reaction of the organ [9]. The latter two processes are of particular importance for the recovery of organ function/structure after the initial damage. The scope of both subjects is extensive; the subject of “Inflammation in AKI” alone could be classified as a separate field of research [21]. Tubulopathy merits particular discussion in this context, particularly within the broader framework of lipid and fatty acid metabolism.

Proximal tubules in the kidneys require significant energy, significantly derived from fatty acid oxidation (FAO) [22], making lipid metabolism a vital area of study in AKI development. However, factors such as ischemia-reperfusion injury, cisplatin, and sepsis can impair FAO, leading to mitochondrial dysfunction and a reduction in the activity of key enzymes involved in lipid metabolism. The resulting deficiencies in FAO hinder ATP production, disrupt cytoskeletal assembly, and contribute to kidney dysfunction and subsequent fibrosis. Early stages of AKI may see an initial increase in FAO [23], but this can transition into a deficiency, leading to lipid accumulation and potential chronic inflammation. AKI is marked by significant lipid accumulation, correlated with the severity of renal injury [24]. While triglycerides are generally nonharmful, excessive free fatty acids (FFAs) and their metabolites can be detrimental. The dysregulation of lipid uptake, biosynthesis, FAO, and efflux often leads to lipid excess in AKI. Lipid accumulation can lead to lipotoxicity, contributing to tubular inflammation and cellular apoptosis, thus exacerbating renal damage and the progression to CKD [25, 26].

Additional alterations in lipid metabolism during AKI include changes in phospholipids and sphingolipids [27, 28]. Phospholipids, essential for biological membranes, often exhibit significant changes that reflect the severity of AKI. Sphingolipids, particularly ceramides, accumulate in renal tissues during AKI and are linked to tubular injury and apoptosis.

Regulation of lipid metabolism in AKI involves various pathways, including the peroxisome proliferator-activated receptor (PPAR) [29], adenosine monophosphate-activated protein kinase (AMPK) [30], farnesoid X receptor (FXR) [31, 32], and sirtuins [33]. These pathways interact intricately, influencing lipid metabolism, oxidative stress, and inflammation. Understanding these regulatory mechanisms is crucial for developing therapeutic interventions for AKI.

### Experimental evidence

The references will be listed according to then date of publication (older to newer).

#### Impairment of endogenous fatty acid metabolism

In 2019, Chiba and colleagues [33] published an interesting study on Sirt(sirtuin)5 deficiency in experimental AKI. Homozygous global Sirt5 knockout (Sirt5-/-) mice (and Sirt5+/- heterozygotes) and littermate wild-type controls (age 10–14 weeks) were studied using renal ischemia–reperfusion injury and cisplatin AKI models, alongside primary mouse and human proximal tubular epithelial cells (PTECs) exposed to hypoxia, combined glucose–oxygen deprivation (CGOD), or cisplatin. Sirt5 deletion caused marked protein lysine hypersuccinylation in kidney, with Sirt5 expression localized to tubular epithelial cells. Despite normal baseline histology and renal function, Sirt5-/- kidneys were protected from ischemic and cisplatin AKI: they showed reduced tubular injury by histology, lower expression of injury markers (Kim-1/Havcr1, Lcn2/NGAL, IL-18), and improved serum creatinine and BUN; Sirt5 knockdown likewise reduced LDH release and injury marker expression in cultured hPTECs. Quantitative succinyl-proteomics identified many Sirt5 target sites concentrated on mitochondrial proteins, with mitochondrial fatty acid oxidation (FAO) the top enriched pathway. Unexpectedly, total FAO measured by 14 C-palmitate oxidation was increased in Sirt5-/- kidneys at baseline and after IRI, even though Complex II mitochondrial respiration was reduced. Further analysis showed that the FAO increase localized to peroxisomes: etomoxir-resistant (peroxisomal) FAO was higher in Sirt5−/− kidney and PTECs, ACOX1 protein and Peroxisomal Membrane Protein 70 abundance were elevated, and peroxisomal FAO was massively increased after severe IRI. Pharmacologic inhibition of peroxisomal FAO (10,12-tricosadiynoic acid) reversed the protective effect of Sirt5 knockdown in hPTECs, supporting a model in which Sirt5 loss drives a shift from mitochondrial toward peroxisomal FAO that underlies tubular and renal protection in AKI. Possible limitations of the study shall be named: The Sirt5-/- mice have a mixed C57BL/6–129 Sv background (with 129 Sv being more resistant to ischemic AKI), which can introduce phenotypic variability despite littermate controls. Kidneys were examined only 7 days after IRI - too short to detect chronic interstitial fibrosis - so long-term effects of Sirt5 deficiency remain unresolved. Recently, Jiang and colleagues [34] designed nitroethylene SIRT5 inhibitors guided by a SIRT5–lead compound co-crystal structure and optimized compound 56 (IC50 = 0.29 µM). Compound 56 improved renal function and histopathology in two mouse models of sepsis-associated acute kidney injury (AKI). Inflammation was reduced, as evidenced by lower serum C-reactive protein (CRP) and decreased renal IL-6, MCP-1, and TNF-α levels, and no significant toxicity was observed. Together, these data support SIRT5 inhibition as a potential therapy for sepsis-associated AKI.

A study published in 2020 [35], also highlighted the significance of fatty acid oxidation in epithelial cells of the proximal tubule, particularly with regard to its heightened susceptibility in AKI. Two models were utilized in the study: cisplatin-induced AKI and 5/6 nephrectomy as a CKD model. The renal expression of stearic acid increased in the AKI model, while that of palmitic acid decreased. Concurrently, the activity of Elovl6, an enzyme that catalyzes fatty acid elongation, exhibited an increase. Utilizing an adenovirus-based approach, Elovl6 was overexpressed in HK-2 cells, leading to an augmentation in the cellular content of C18:0 (stearic acid), which was concomitant with elevated levels of cell damage. Concurrently, the researchers observed a heightened cytotoxic potency for C18:0 in comparison to C16:0 (palmitic acid) in dilution experiments. The enhanced toxicity of C18:0 was further substantiated in ER stress analyses, as evidenced by the elevated transcription rate of the ER stress marker CHOP, which exceeded the level induced by C16:0. Subsequent experiments centered on the significance of Advanced Oxidation Protein Products (AOPP) in the process of Elovl6 activation in CKD. In a supplementary Elovl6 knockout model, the kidney-damaging potency of the enzyme in CKD was then demonstrated. With regard to AKI, the study enabled the identification of stearic acid as a mediator of tubule cell damage in all cases. The study’s findings indicated that stearic acid plays a pivotal role in the pathogenesis of tubule cell damage in AKI. There were nevertheless some limitations: the study used immortalized HK-2 proximal tubule cells, which may differ from primary human cells (e.g., in transport or metabolic responses). Free fatty acids (palmitic acid C16:0 and stearic acid C18:0) were applied at 100–500 µM, but it’s unclear if such levels occur in vivo at interstitial or intracellular sites.

An experimental study published in 2020 by Li et al. [36] focused on the potential AKI-protective effect of sirtuin 3 (Sirt3). Using cisplatin (20 mg/kg, ip) to induce acute kidney injury (AKI) in male mice and cultured mouse renal tubular epithelial cells, the study combined biochemical, histological, metabolomic, and molecular assays to examine fatty acid oxidation (FAO), mitochondrial function, and the role of Sirt3. Seventy-two hours after cisplatin, mice showed AKI with elevated serum creatinine and BUN, proximal tubular injury, marked renal lipid accumulation (oil red O), higher free fatty acids (notably palmitic acid ~ 19-fold), increased ROS and lipid peroxidation (4HNE), decreased GPX4, and reductions in TCA-cycle metabolites and ATP. FAO-related proteins (PPARα, CPT1A, ACADL) and ACADL activity were decreased in AKI, and palmitate exposure induced apoptosis in tubular cells. Sirt3 expression was reduced by cisplatin; Sirt3 knockout (KO) mice developed worse kidney dysfunction, greater lipid accumulation, lower FAO protein levels and activity, more apoptosis, and more severe mitochondrial damage than wild-type (WT) mice. Pre-treatment with honokiol (HKL), a Sirt3 agonist, increased Sirt3 expression, restored FAO protein levels, reduced FFA accumulation and tubular injury, improved ATP production and mitochondrial morphology, and lowered mitochondrial ROS and 4HNE in WT but not in Sirt3 KO mice. Mechanistically, Sirt3 appears to deacetylate LKB1, promoting LKB1–AMPKα activation (increased p-AMPKα and p-ACC) to stimulate FAO and protect mitochondria and energy metabolism in cisplatin-induced AKI. Two possible limitations of the study should however be adressed: (I) use of palmitate to model lipotoxicity - in the cell experiments, mouse renal tubular epithelial cells were incubated with 0.2 mmol/L palmitic acid to induce cellular toxicity. In vivo, however, fatty acids rarely occur in isolation: they are typically bound to transport proteins (e.g., albumin) or present as complex mixtures, which substantially alter both toxicity and cellular uptake. (ii) off-target effects of honokiol (HKL) - HKL was used in the study as a Sirt3 activator and showed protective effects in wild-type animals. Although the authors convincingly demonstrate Sirt3 dependence using Sirt3 knockout mice (HKL is ineffective in the knockouts), honokiol is a natural product with known pleiotropic actions. It exerts intrinsic antioxidant effects and modulates the cytoskeleton, factors that cannot be entirely excluded as pharmacological confounders in the wild-type background. Lately, Wang and colleagues [37] targeted Sirt3 under experimental AKI conditions. Using CLP mice, LPS-stimulated HK-2 cells, and scRNA-seq, the study showed sepsis as inductor of metabolic reprogramming in renal epithelial cells with mitochondrial dysfunction and suppression of OXPHOS. Ethyl pyruvate (EP) combined with AMPK activation (AICAR) restored NAD+/NADH balance, mtDNA, respiratory chain activity, and fission–fusion dynamics, enhanced autophagy and reduced apoptosis via the AMPK–mTOR–SIRT3 pathway. It finally improved renal outcomes - effects partly reversed by SIRT3 inhibition, supporting EP+AMPK activation as a metabolism-targeted therapy for SA-AKI.

In 2021, Li and colleagues [38] used C57BL/6 mice (including Fabp4 - fatty acid-binding protein 4 - knockout) and multiple acute and chronic kidney injury models - folic acid (FA; single 250 mg/kg IP, analyzed 48 h post), aristolochic acid (AA; 5 mg/kg IP daily ×4, analyzed day 5), glycerol-induced rhabdomyolysis, ischemia–reperfusion (30 min clamp, 24 h reperfusion), and unilateral ureteral obstruction (UUO) - to investigate FABP4’s role in renal injury. RNA-seq and follow-up assays showed Fabp4 expression was markedly increased in injured kidneys (≈ 3-fold in some AKI models) and localized to tubular epithelial cells. Pharmacologic inhibition (BMS309403, 40 mg/kg/day) or genetic deletion of Fabp4 reduced serum creatinine and BUN, lessened tubular damage, and decreased collagen deposition and fibrotic markers (α-SMA, Fn, Col1, Col4). Both interventions also lowered proinflammatory cytokine mRNAs (IL-6, MCP1, TNF-α). Mechanistically, Fabp4 deletion or inhibition restored PPARγ expression and suppressed NF-κB p65 and STAT3 activation (reduced phosphorylation). In HK-2 cells, LPS increased FABP4 and fibrotic/inflammatory markers; BMS309403 dose-dependently reduced FABP4, enhanced PPARγ, and inhibited p-p65 and p-STAT3, with pioglitazone producing similar antiinflammatory effects while the PPARγ antagonist did not. Overall, elevated FABP4 contributes to kidney injury, inflammation, and fibrosis, and targeting FABP4 protects against renal damage - likely via PPARγ upregulation and inhibition of NF-κB/STAT3 signaling. The study was limited by use of only 8-week-old male mice (reducing generalizability to females), assessment of only early fibrosis (48 h in the FA model and day 5 in the AA model) so long-term CKD effects weren’t evaluated, and reliance on abrupt, high-dose toxin models (single 250 mg/kg folic acid or 5 mg/kg aristolochic acid over four days) that did not reflect gradual, multifactorial human kidney disease. A newer study from 2026 [39] found FABP4 markedly upregulated in plasma from SA-AKI patients and in kidneys of SA-AKI model mice; pharmacologic inhibition with BMS-309,403 improved renal function, reduced tubular injury and inflammation in LPS-treated mice and prevented RSL3-induced ferroptosis in HK-2 cells. These protective effects correlated with reduced lipid peroxidation and iron deposition, improved mitochondrial function, and normalization of GPX4 and ACSL4, supporting that FABP4 promotes ferroptosis in SA-AKI and that targeting FABP4 may be a novel therapeutic strategy.

The functional significance of Krüppel-like factor 15 (KLF15), a zinc-finger rich transcription factor, in the process of aristolochic acid I (AAI) nephropathy was shown by Piret et al. in 2021 [40]. Using proximal tubule (PT)-specific Klf15 knockout mice (Klf15 PTKO, generated with Pepck-Cre) and multiple kidney injury models (aristolochic acid I [AAI]-induced AKI with remodeling, unilateral ischemia–reperfusion with uninephrectomy, and UUO), the authors showed that KLF15 expression in PT cells is reduced after injury and that PT-specific loss of KLF15 (~ 60% reduction) worsens acute injury and promotes fibrosis. KLF15 PTKO mice had larger rises in serum creatinine and urea, greater PT loss, inflammation, myofibroblast proliferation, and increased collagen I and α-SMA deposition in both the active and remodeling phases after AAI, and showed impaired recovery after IRI. RNA-seq of cortex revealed 3,042 differentially expressed genes: immune and integrin/focal adhesion pathways were upregulated in Klf15 PTKO mice (with a biphasic immune activation), while metabolic pathways - particularly fatty acid oxidation (FAO), PPAR signaling, and amino acid/carbohydrate metabolism - were downregulated even at baseline. Bioinformatic and experimental data implicate cooperative regulation of FAO genes by KLF15 and PPARα: Ppara, Cpt1a, and Acaa2 were reduced in Klf15 PTKO cortex and in primary PT cells after KLF15 5 deletion; ChIP in KLF15-overexpressing cells validated KLF15 binding at CPT1A and ACAA2 promoters, often adjacent to PPARα sites in open chromatin. Functional assays showed that loss of KLF15 impaired palmitate-driven oxygen consumption in primary PT cells, while KLF15 overexpression in HK-2 cells partially preserved FAO after AAI. Human kidney samples from CKD patients exhibited reduced nuclear PT KLF15 and AQP1 mislocalization; tubulointerstitial expression data demonstrated positive correlations between KLF15 and PPARA and independent associations of both KLF15 and PPARA with eGFR in multivariate modeling. Together, the data support a model in which PT KLF15 cooperates with PPARα to maintain FAO and metabolic resilience, and its loss predisposes to heightened inflammation and fibrosis after kidney injury. Two limitations of the study should be briefly noted: (I) - incomplete in vitro rescue: live-cell metabolism assays showed that overexpression of KLF15 alone did not fully restore fatty acid oxidation (FAO) after aristolochic acid I (AAI)–induced injury. This suggested that KLF15 does not act autonomously but depends on cofactors or other transcription factors (e.g., PPARα), which complicates the translational therapeutic approach. (ii) - lack of in vivo evidence for therapeutic reversibility: although KLF15 knockout was shown to worsen injury, the study provided no in vivo data demonstrating therapeutic rescue (for example, inducible KLF15 overexpression or use of KLF15 activators after injury). A recent integrative multi-omics analysis [41] also identified Krüppel-like factor 15 (KLF15) as a top downregulated transcription factor in cisplatin-induced acute kidney injury, with single-cell data showing proximal tubule cells most affected. KLF15 loss was linked to inflammation, apoptosis, and suppressed metabolic pathways (TCA cycle, fatty acid oxidation), was validated in a mouse model (20 mg/kg IP, 72 h), and virtual screening nominated six FDA-approved drugs (including simeprevir, lomitapide, and Avodart) as potential KLF15-targeting compounds.

Xiong et al. [42] analyzed the role of UCP1, a member of the superfamily of uncoupling proteins (UCPs) related to energy metabolism, in a model of cisplatin-induced AKI. The study examined the connection between lipid accumulation, UCP1 expression, and acute kidney injury (AKI). The findings indicated a correlation between AKI and significant lipid accumulation, particularly triglycerides, with a positive correlation to the severity of kidney injury. Principal component analysis confirmed the reliability of the experimental models, and lipid metabolomics revealed distinct differences between cisplatin-induced AKI mice and control mice. UCP1, a crucial uncoupling protein for lipid degradation, was significantly downregulated in AKI and negatively correlated with the severity of kidney injury. The expression of UCP1 was primarily localized in renal tubules, and its levels decreased as kidney damage progressed. It was demonstrated that increasing UCP1 expression, either through lentiviral overexpression or the UCP1 agonist CL316243, reduced lipid accumulation in AKI models and alleviated renal injury markers, inflammation, and apoptosis. The study also revealed that upregulating UCP1 activated the AMPK/ULK1/autophagy pathway, promoting autophagy and thus improving cellular function in AKI. In both in vitro and in vivo experiments, UCP1 overexpression led to significant improvements in kidney morphology and function, suggesting that enhancing UCP1 expression could be a potential therapeutic strategy to mitigate lipid accumulation and its detrimental effects in AKI. The findings underscore the pivotal role of UCP1 in lipid metabolism and its capacity to influence the progression of AKI through autophagy activation. A potential limitation of the study is briefly noted: CL316243 is highly selective for the β3-adrenergic receptor, which upregulates UCP1 expression. However, systemically administered adrenergic agonists can cause widespread metabolic and cardiovascular side effects in vivo in mice - for example, systemic lipolysis in adipose tissue - that may indirectly influence the kidney rather than acting exclusively within the renal tubules. Further data on the AKI protective role of UCP1 comes from a study by Bao et al. (2026 [43]). Baicalin (a plant-derived compound with potent antioxidant and anti-inflammatory properties) protected against sepsis-associated acute kidney injury by activating the PPAR-γ/UCP1 signaling pathway: in LPS-induced mice and HK-2 cells it reduced inflammation, oxidative stress, and apoptosis, restored mitochondrial function, upregulated PPAR-γ/UCP1 by RNA-seq, showed stable docking with UCP1, and lost protective effects when PPAR-γ or UCP1 were knocked down.

Wang et al. [44] also investigated the role of fatty acid-binding protein 4 (Fabp4) in septic acute kidney injury (AKI) using a model of cecal ligation and puncture (CLP) in mice. The study found that Fabp4 expression was significantly elevated in the kidneys of CLP-induced septic AKI mice, correlating with increased inflammation and apoptosis markers. Genetic deletion of Fabp4 (Fabp4 KO) or pharmacological inhibition using BMS309403 led to improved kidney function and reduced kidney injury, inflammation, and apoptosis in CLP mice. The upregulation of Fabp4 in septic AKI has been linked to the activation of TLR4/MyD88/JNK/c-Jun signaling pathways. Both Fabp4 KO and BMS309403 treatment effectively suppressed the expression of key inflammatory and apoptotic markers. In vitro studies using TCMK-1 cells confirmed that Fabp4 inhibition reduced inflammation and apoptosis induced by LPS, further supporting the role of Fabp4 in mediating these processes. The study also identified a positive feedback loop between Fabp4 and c-Jun activation, suggesting that Fabp4 amplifies inflammatory responses and apoptosis in septic AKI. Finally, the researchers created a mouse model with renal tubular epithelial cell-specific deletion of Fabp4, demonstrating that this specific deletion mitigated CLP-induced septic AKI, confirming the critical role of Fabp4 in the pathogenesis of septic AKI. Overall, the findings indicate that targeting Fabp4 could be a potential therapeutic strategy for septic AKI. One potential limitation of the study should be briefly noted: although the authors utilize both genetic knockout models and the chemical inhibitor BMS309403 to block FABP4, it is well-established that BMS309403 can exhibit potential off-target effects (e.g., minor cross-reactivity with other fatty acid-binding proteins such as FABP3 or FABP5). Consequently, complete specificity of the compound is not guaranteed in vivo.

Another study from Chiba et al. [45] was published in 2024. It evaluated long-chain acyl-CoA dehydrogenase (LCAD). This study investigatied how the genetic deletion of long-chain acyl-CoA dehydrogenase (LCAD), a key enzyme in mitochondrial FAO, affects the kidney’s response to acute damage. The researchers utilized two distinct in vivo experimental models of AKI: cisplatin-induced nephrotoxicity and ischemia-reperfusion injury (IRI). LCAD-/- mice were significantly protected against both cisplatin- and IRI-induced AKI. They exhibited preserved renal function and substantially less structural tubular damage compared to WT animals. In addition, kidneys showed a dramatic reduction in lipid peroxidation, lower reactive oxygen species (ROS) production, and decreased cell death. Ferroptosis-associated pathways were significantly attenuated. Finally, while WT proximal tubules suffered a catastrophic drop in mitochondrial respiration during AKI, LCAD-/- cells maintained their oxygen consumption rates and mitochondrial structural integrity. It was proposed that mitochondrial LCAD loss triggers the upregulation of peroxisomal fatty acid oxidation (via pathways resembling PPARα activation). Peroxisomal FAO compensates for the energy deficit without generating the high levels of mitochondrial ROS typically seen during AKI. The study has nevertheless some limitations: peroxisomal FAO was inferred indirectly using etomoxir (which has off-target effects) rather than measured directly; and only a constitutive LCAD knockout was tested rather than transient pharmacologic inhibition. The Sirt5–LCAD axis was not directly examined, IRI experiments used only males, ferroptosis assessment relied on non-specific TUNEL plus few mRNA markers, some groups had very small n and mixed genetic background, and only short-term outcomes were reported.

### Exogenous modulation of fatty acid metabolism

The first study to be named was published by Hassan et al. in 2009 [46]. It examined the impact of dietary omega-3 polyunsaturated fatty acids (PUFAs) on renal ischemia-reperfusion injury (IRI) in a mouse model. Prolonged ischemia was associated with increased inflammation, as indicated by elevated polymorphonuclear (PMN) cell infiltration and serum creatinine levels. The expression of heme oxygenase-1 (HO-1), a stress response protein, increased with longer ischemic durations. The analyses further explored the impact of an acute dietary increase in omega-3 PUFAs, using menhaden oil, compared to a diet high in omega-6 PUFAs from corn oil. The results showed that mice on the diet with more omega-3 PUFAs had better kidney function and survival rates after ischemic events. Only severe ischemia led to higher levels of serum creatinine. In contrast, the group receiving an omega-6 PUFA diet exhibited high mortality and increased renal inflammation. Omega-3 PUFAs significantly reduced PMN infiltration and levels of inflammatory cytokines and chemokines following IRI. Additionally, the study found that the diet containing omega-3 PUFAs led to a decrease in the production of harmful eicosanoids and an increase in the endogenous formation of protective DHA-derived autacoids, such as PD1 and 17-HDHA. The study also highlighted that systemic treatment with these DHA-derived autacoids reduced PMN recruitment and enhanced renal HO-1 expression, both in injured and healthy kidneys. In vitro experiments confirmed that these autacoids can increase HO-1 expression in mesangial cells. The findings suggested that dietary omega-3 PUFAs offer significant protection against renal IRI, likely through the modulation of inflammatory responses and enhancement of cytoprotective pathways. If limitations of the investigation are to be noted, the following points should be considered: animals were sacrificed and tissue/blood samples were collected only at 24 h post-ischemia. While this time point captures the peak acute inflammatory response and the initial decline in glomerular filtration rate (GFR), the study provides no data on longer-term survival, chronic structural remodeling, or the potential progression to fibrotic kidney disease in the surviving omega-3 cohort. Extending ischemia to 45 min resulted in 100% mortality in the omega-6 group versus 0% in the omega-3 group. Because the omega-6 animals died rapidly, direct comparison of structural and molecular repair mechanisms between groups over an extended timeline was not possible.

A 2017 study by Deng et al. [47] evaluated the effects of two major fatty acid metabolites on acute kidney injury in a murine ischemia-reperfusion model. The project focused on the major epoxide metabolite of omega-3 polyunsaturated fatty acid (PUFA) docosahexaenoic acid, ‘19 [20]-EDP’ and the major epoxide metabolite of omega-6 PUFA arachidonic acid, ‘14 [15]-EET’. Docosahexaenoic acid (DHA), for example, was documented to exert antiproteinuric effects in a cat CKD model [48], whereas arachidonic acid (ARA) has been discussed as a potential mediator of the AKI-to-CKD transition [49]. In the present study, the two metabolites exhibited de facto opposing effects on renal function and morphology in the AKI model. Specifically, 14 [15]-EET was found to have a protective effect on renal tissue, while 19 [20]-EDP exacerbated the initial kidney injury. Renal function was quantified using multidimensional methods, including plasma urea nitrogen and creatinine levels, as well as renal NGAL contents. The in vivo findings could be reproduced in vitro (murine renal tubular epithelial cells - mRTECs). The metabolite 14 [15]-EET was observed to reduce the ischemia/reperfusion-associated reduction of GSK3β (glycogen synthase kinase 3β) phosphorylation and the extent of apoptosis of cultured mRTECs under hypoxia. On the one hand, the study identified a novel nephroprotective fatty acid metabolite in AKI (14 [15]-EET); on the other hand, it revealed the complexity of the influence of structurally related fatty acid derivatives on renal function and structure. One minor limitation needs to be adressed: in vitro cell culture data clearly demonstrated that 19 [20]-EDP dose-dependently reduced GSK3β phosphorylation in mRTECs. However, when administered in vivo, 19 [20]-EDP alone failed to significantly alter or decrease GSK3β phosphorylation in the actual kidney tissue.

An experimental study from 2022 [50] evaluated the nephroprotective effect of oleic acid in the murine model of LPS-induced AKI. Components of the sticky Chinese foxglove (Rehmannia glutinosa) are used in Chinese medicine to treat kidney diseases, a major component of which is oleic acid. In vivo, OA improved LPS-induced renal histopathology, lowered serum creatinine and urea, normalized macrophage, granulocyte, and neutrophil proportions, reduced proinflammatory cytokines (IL-2, TNF, IFN-γ), increased IL-10, and decreased iNOS, COX-2, and NF-κB p-p65. OA also reduced oxidative stress by lowering ROS and inhibited apoptosis by reducing caspase-9/3 activation and Bax while increasing Bcl-2. In NRK-52e cells, OA decreased ROS and intracellular Ca2+, increased mitochondrial membrane potential, and upregulated Ras, Raf1, SHC, and PPAR-γ. These effects were blocked by the Ras inhibitor FTS, supporting the involvement of Ras/MAPK/PPAR-γ signaling. Metabolomic analysis showed that OA-treated groups clustered close to controls and identified 30 differential serum biomarkers and 10 altered pathways, including phenylalanine, purine, sphingolipid, and taurine/hypotaurine metabolism. Overall, OA alleviated LPS-induced AKI through anti-inflammatory, antioxidant, and anti-apoptotic effects associated with the Keap1/Nrf2 and Ras/MAPK/PPAR-γ pathways, as well as metabolic regulation. As explicitly stated by the authors in the discussion section, a major shortcoming of this project was that it failed to compare the in vivo therapeutic effects of oleic acid (OA) against a validated positive control/reference drug for acute kidney injury (AKI). For the complex protein expression analyses, the Western blot tracking of crucial apoptotic, MAPK, and PPAR-γ components was limited to a sample size of *n* = 3 mice per group. Small sample sizes dramatically elevate statistical variance and restrict the generalizability of the reported pathway transformations.

In 2022, Shi et al. [51] investigated the effects of different dietary substances on kidney function and inflammation pathways in mice with acute kidney injury (AKI) induced by cisplatin. The treatment with cisplatin resulted in substantial weight reduction (14.8%), reduced food intake, aberrant behavioral changes, and a decrease in liver weight of 25.5%. Kidney function was adversely affected, with BUN increasing by 2.31 times and Cr by 1.03 times compared to the normal group. Dietary interventions, particularly with Cur-2DHA (Docosahexaenoic acid-acylated curcumin diester), led to a significant reduction in BUN (71.7%) and Cr (48.1%). Histopathological analysis revealed severe kidney damage from cisplatin, including necrosis and inflammatory infiltration. However, Cur-2DHA significantly alleviated this damage. Oxidative stress was also assessed, revealing decreased glutathione (GSH) levels and increased malondialdehyde (MDA) in the model group. Cur-2DHA and Cur-DHA (Docosahexaenoic acid-acylated curcumin monoester) improved GSH levels and reduced MDA. The study further explored the NLRP3 inflammasome signaling pathway, finding that cisplatin elevated pro-inflammatory cytokines such as TNF-α, IL-1β, and IL-6. Cur-2DHA significantly inhibited these cytokines and the activation of NLRP3-related proteins. Furthermore, the fatty acid composition in the kidneys was affected by cisplatin, with alterations in specific fatty acid ratios. The incorporation of Cur-2DHA through dietary supplementation led to enhancements in the fatty acid profile within the kidneys. The findings indicated that Cur-2DHA and related compounds may offer protective benefits against cisplatin-induced AKI through various mechanisms, including the reduction of inflammation and oxidative stress, and the stabilization of specific fatty acid ratios. It nevertheless needs to be mentioned, that mice were pre-treated with the dietary interventions for 7 days *before* receiving cisplatin injections to induce AKI. In a real-world clinical environment, AKI is mostly unpredictable or diagnosed after the injury has occurred. The study demonstrates a preventative or prophylactic effect rather than a therapeutic treatment post-injury.

Shan et al. [52] investigated the effects of exogenous administration of docosahexaenoic acid (DHA), on kidney function and structure in two AKI models (rhabdomyolysis- and folic acid-induced AKI (Rha-AKI and FA-AKI)). The focus of the study was the modulation of ferroptosis [53]. The reason for the supply of DHA was the already known assumption that increased peroxidation of PUFA could be involved in the pathogenesis of AKI. Mice received 7-day dietary supplements (3% w/w) of DHA-rich fish oil, oleic-acid–rich camellia seed oil, or lard (SFA). a Fer-1 (ferrostatin-1) group was given an antioxidant ferroptosis inhibitor. In vivo, Rha-AKI caused marked weight loss, severe tubular damage and fibrosis, elevated BUN/creatinine, and hyperlipidemia. FA-AKI produced milder, more transient injury. Fer-1 protected against FA-AKI but not Rha-AKI, indicating ferroptosis was involved in FA-AKI only. DHA accumulated in kidney and, in Rha-AKI, improved kidney function, reduced histologic injury, lowered lipid-peroxidation markers (MDA, 4-HNE) and inflammatory mediators, and mitigated hyperlipidemia. By contrast, DHA (and to a lesser extent OA) worsened FA-AKI: mice given DHA showed greater tubular loss, higher BUN/creatinine trends, increased MDA and 4-HNE aggregation, elevated inflammatory gene expression, and poorer recovery with more fibrosis - suggesting higher CKD risk. In HK-2 cells, DHA did not alter myoglobin (Fe2+)-mediated cytotoxicity (which was not ferroptotic), but it exacerbated ferroptosis induced by RSL3/erastin. This enhancement was reversible by Fer-1. Overall, DHA has context-dependent effects: protective in rhabdomyolysis injury but promotive of ferroptosis-related damage in folic acid–induced AKI. Limitation: clinical acute kidney injury in humans is rarely so clear-cut; real patients frequently present with overlapping etiologies where multiple cell death pathways (apoptosis, necroptosis, and ferroptosis) coexist simultaneously.

A 2024 investigation [54] examined the protective effects of oleoylethanolamide (OEA) on kidney function impaired by folic acid (FA) in mice. The OEA treatment resulted in the normalization of kidney dysfunction induced by FA, as evidenced by reduced serum creatinine and urea levels. Furthermore, OEA led to a reduction in albumin excretion. Histological analysis revealed severe tubular damage in mice treated with FA, which was significantly alleviated by OEA. The treatment also reduced inflammation markers and the expression of tubular injury markers, suggesting significant renoprotective effects of the substance. Furthermore, OEA exhibited a capacity to curtail renal fibrosis, as evidenced by diminished expression of fibrosis-related genes and a reduction in collagen deposition. The underlying mechanism by which OEA exerted its effects involved the activation of the Peroxisome-Proliferator-Activated Receptor (PPAR)-α receptor, which is responsible for regulating the β-oxidation process in peroxisomes. This hypothesis was confirmed through experiments with PPAR-α knockout mice, where OEA failed to ameliorate kidney damage. In human proximal tubular HK-2 cells, OEA inhibited TGF-β1-induced epithelial-to-mesenchymal transition (EMT) and fibrosis markers through a PPAR-α-dependent mechanism. In summary, OEA has demonstrated potential as a therapeutic agent for kidney injury and fibrosis by modulating inflammatory responses and fibrotic pathways. The authors noted that while OEA’s benefits were largely absent in PPAR-α knockout mice, there was still a non-significant trend toward a reduction in inflammatory and fibrotic markers. They acknowledge that alternative pathways - such as transient receptor potential vanilloid 1 (TRPV1) or GPR119, both of which bind OEA - could be contributing to the protective effects, but these were not experimentally isolated or controlled.

Silva and colleagues [55] addressed an essential, nephroprotective, endogenous mechanism, fatty acid oxidation in peroxisomes. Wild-type male mice were fed diets supplemented with medium- and long-chain dicarboxylic acids (notably DC8 and DC12) and subjected to two models of acute kidney injury (AKI): unilateral renal ischemia–reperfusion injury (IRI) with delayed contralateral nephrectomy, and a single-dose cisplatin nephrotoxicity model. DC10 and DC12 produced widespread protein lysine succinylation in liver and kidney, whereas DC8 induced marked hypersuccinylation specifically in kidney; succinylation peaked around 72 h of feeding and largely washed out by five days. DC8 supplementation (dose-response showed 5% w/w ≈ 10% w/w efficacy; 2.5% suboptimal) strongly protected against both IRI- and cisplatin-induced AKI: lower serum creatinine and BUN, reduced NGAL expression, preserved tubular morphology, fewer TUNEL-positive cells, and no weight effects. Global proteomics (DIA-MS) quantified ~ 4,000 proteins and > 4,500 succinylated peptides and revealed that IRI caused large losses of mitochondrial and peroxisomal proteins and mitochondrial succinylation; DC8 blunted mitochondrial protein loss, preserved complex I subunits, and prevented loss of complex I–driven respiration. In contrast, DC8 massively increased peroxisomal protein succinylation and abundance - particularly on core peroxisomal fatty-acid–oxidation (FAO) enzymes - and preserved peroxisomes after IRI. Functional assays (radiolabeled FAO with etomoxir discrimination and 14 C-peroxisomal substrates) and immunofluorescence confirmed increased peroxisomal FAO and greater intact peroxisome abundance in DC8-fed kidneys. Overall, kidney-targeted metabolic remodeling by DC8 - marked by enhanced peroxisomal FAO, peroxisomal protein succinylation, and preservation of mitochondrial function - correlated with robust protection from multiple forms of AKI. Two limitations need to be adressed: (I) dicarboxylic acid (DCA) supplementation was administered 7 days before the induction of acute kidney injury (AKI). In clinical scenarios, patients often present with AKI after the insult has already occurred, meaning this study does not establish whether can work as a treatment or rescue therapy post-injury. (ii) The most effective dosing tested was a 5% to 10% w/w supplementation mixed into a mush diet. Translating a 5%–10% daily dietary weight requirement from mice to humans represents an incredibly high mass volume, which could pose severe logistical and palatability challenges for human clinical trial formulations.

Tokumaru and colleagues [56] investigated the effects of omega-3 polyunsaturated fatty acids (omega-3PUFAs) on kidney health, particularly in the context of acute kidney injury (AKI) transitioning to chronic kidney disease (CKD). Mice were fed diets with high levels of omega-3 PUFAs (linseed oil) or low levels of omega-3 PUFAs (soybean oil) for a period of four weeks prior to the induction of renal ischemia-reperfusion (IR) injury. The results demonstrated that mice consuming the linseed oil diet exhibited significantly higher levels of omega-3 PUFAs, particularly eicosapentaenoic acid (EPA), in their renal tissues compared to those consuming the soybean oil diet. Subsequent to undergoing ischemic injury, the linseed oil group demonstrated enhanced survival rates and diminished renal damage, as indicated by diminished levels of blood urea nitrogen (BUN) and serum creatinine, as well as a reduction in tubular injury and fibrosis. The antifibrotic effects of omega-3 polyunsaturated fatty acids (omega-3PUFAs) were further confirmed in a unilateral ureteral obstruction (UUO) model, where the linseed oil diet also reduced fibrosis and myofibroblast marker expression. Metabolomic analysis revealed that the linseed oil diet led to increased levels of beneficial eicosapentaenoic acid (EPA) metabolites while decreasing levels of arachidonic acid (AA) metabolites. Key metabolites such as 18-hydroxyeicosapentaenoic acid (18-HEPE) and 17,18-epoxyeicosatetraenoic acid (17,18-EpETE) were identified as potentially responsible for the antifibrotic effects of EPA. These metabolites were shown to inhibit the expression of α-smooth muscle actin (α-SMA) in cultured rat renal fibroblasts, suggesting a mechanism through which omega-3 polyunsaturated fatty acids (omega-3PUFAs) exert their protective effects on kidney tissue. In summary, the findings indicate that a diet enriched with omega-3 PUFAs can enhance survival and mitigate renal damage and fibrosis following acute kidney injury (AKI), highlighting the therapeutic potential of these fatty acids in renal health. One limitation should finally be adressed: the authors note that 18-HEPE is known to be a precursor for Resolvin E1, a highly potent anti-inflammatory and antifibrotic lipid mediator. However, due to the technical boundaries of their methodology, the liquid chromatography-mass spectrometry (LC-MS) analysis “failed to detect the presence of resolvins” in the renal tissues. This leaves the precise downstream metabolic pathway partially unverified.

As the title of this article already indicates, all of the studies discussed are experimental in nature. We recently published a review article on the therapeutic use of lipid components in human CKD and AKI [57]. We will briefly present two studies from that article, in part to illustrate the translational options that may arise from experimental data. Hoogeveen et al. [58] examined 2,344 patients after myocardial infarction (mean age 69 years; 81% men) to assess the effects of marine (EPA/DHA) and plant (ALA) n-3 fatty acids on renal function over a median follow-up of 41.3 months. Baseline characteristics included 19% diabetes, 23% obesity, and 15% current smokers; median fish intake was 14.7 g/day and 5.2% used fish-oil supplements. The EPA-DHA group received on average 239 mg EPA + 159 mg DHA per day; the ALA group received 1.99 g ALA daily. After 40 months, eGFR fell by 6.9 ml/min/1.73 m² in the placebo group versus 2.1 ml/min/1.73 m² in the EPA-DHA group - a roughly 30% relative attenuation of decline; ALA produced no significant effect. The treatment effect was larger in patients with preexisting CKD (between-group difference 4.9 ml/min/1.73 m²). The incidences of new CKD and rapid renal function loss were 15% and 34%, respectively, and odds ratios did not demonstrate a clear advantage for EPA-DHA over placebo. Overall, EPA-DHA modestly slowed eGFR decline, with effects that varied by CKD status.

In a separate comparison of omega-3 versus N-acetylcysteine (NAC) in CKD patients undergoing coronary angiography [59], the NAC group was older (66.18 vs. 60.65 years; *p* = 0.006) and had a higher prevalence of hyperlipidemia (38.8% vs. 17.5%; *p* = 0.007); ACE inhibitor/ARB use was more common in the omega-3 group (54.0% vs. 34.3%; *p* = 0.024). eGFR, diabetes, hypertension, and prior cardiac disease were similar between groups. The overall rate of contrast-induced nephropathy was 7.7% (*p* = 0.919) and did not differ between groups. Rates of renal replacement therapy (8 patients; not significant), in-hospital deaths (6; *p* = 0.361), peri-procedural MI/stroke, and length of stay (6.7 vs. 6.16 days; *p* = 0.714) also showed no significant differences. In summary, no clear advantage of one treatment over the other was observed.

**Fig. 2:**
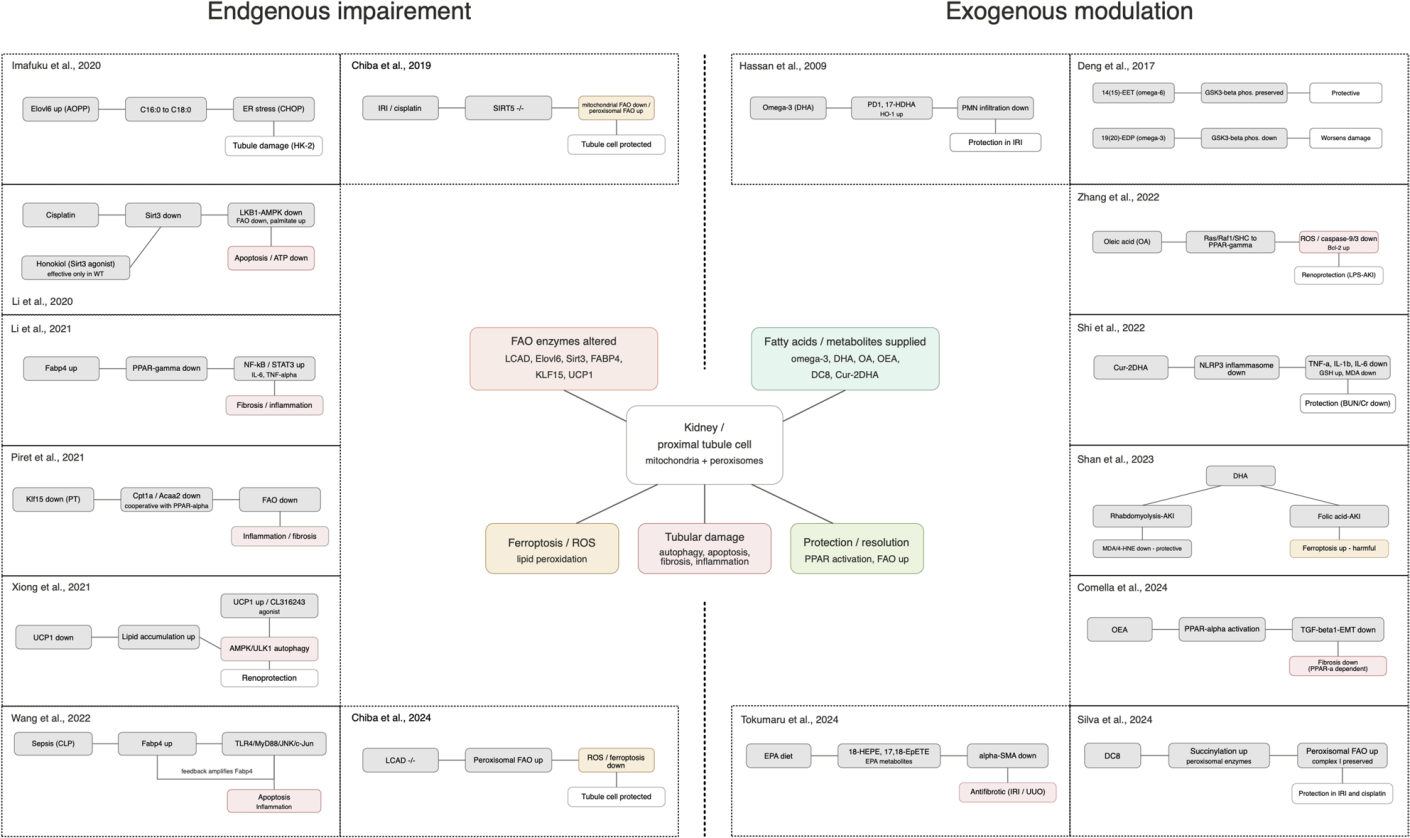
Graphical summary of the cited studies. The central diagram depicts factors involved in the endogenous modulation of fatty acid oxidation that were analyzed in the cited studies (“FAO enzymes altered” – light red background). In addition, the process of ferroptosis and the increased formation of reactive oxygen species (ROS) are illustrated (“Ferroptosis / ROS” – light brown background). Finally, at the bottom center is a list of processes associated with tubular cell damage (“Tubular damage – autophagy, apoptosis, fibrosis, inflammation” – pink background). The majority of the studies discussed were each presented using a separate flowchart. The last (or sometimes second-to-last) box of each flowchart corresponds in color to the processes in the central figure. The same approach was taken for the studies on exogenous modulation (to the right of the dotted center line)

Table 1; Fig. 2 summarize the main findings of all the studies presented, including the reference, design and outcome of each study.

## Discussion

In general, mouse models have important limits for translating findings to humans. Anatomical and physiological differences (very high mouse heart rate, different cardiac electrophysiology, much thinner joint cartilage with different biomechanics) limit applicability. In principal, mouse diseases are artificially induced (genetic, chemical, surgical) and do not capture humans’ multifactorial pathogenesis. In addition, inbred laboratory strains lack the genetic and environmental heterogeneity of human patients. Although mice and humans share about 85% of protein-coding genes, gene regulation and expression often differ; the immune systems are fundamentally different (e.g., lymphocyte-to-neutrophil ratios and cytokine responses), and metabolic/pharmacokinetic differences - such as a roughly sevenfold higher metabolic rate and distinct cytochrome P450 activity - change drug efficacy and toxicity. Mice’ short lifespan (≈ 2–3 years) makes modeling chronic, age-related diseases difficult. Finally, controlled lab environments - specific-pathogen-free housing and sub-thermoneutral temperatures (~ 30 °C is thermoneutral) - alter immunity and metabolism, introducing additional confounders that reduce translatability. Therefore, any findings from mouse models regarding disease onset, progression, and treatment should be interpreted with great caution when extrapolating to humans. Nonetheless, these models yield valuable insights into pathogenic mechanisms, and a complete replacement of animal models with alternative experimental strategies has not yet been achieved. Promising advances, however, come with the broader availability of so-called organoids - tissues or organs generated in vitro [60]. which already demonstrate remarkable functional and structural similarities to real tissue. Only a few references on kidney organoid models are cited here; their contents will not be discussed in detail [61–64].

### Impairment of endogenous fatty acid metabolism

A central paradigm emerging from the work of Chiba et al. [33] and Silva et al. [55] is a stress-induced functional shift between mitochondrial and peroxisomal fatty acid oxidation (FAO). Under ischemia–reperfusion injury (IRI) or cisplatin toxicity, mitochondrial FAO collapses due to loss of critical enzymes (e.g., LCAD/ACADL), driving massive mitochondrial ROS production. In contrast, compensatory activation of peroxisomal FAO appears to provide a protective alternative. Chiba et al. [33] showed that genetic loss of LCAD paradoxically protects against acute kidney injury (AKI) by upregulating peroxisomal β-oxidation through PPAR-alpha-like signaling. Peroxisomal β-oxidation generates far fewer toxic oxygen radicals than the mitochondrial respiratory chain, thereby attenuating downstream ferroptosis. Silva et al. [55] complement this axis by demonstrating that dietary induction of peroxisomal function with dicarboxylic acids (DC8) robustly protects the organ from mitochondrial dysfunction.

Epigenetic and transcriptional regulators further modulate these metabolic responses. Li et al. [36] found that Sirtuin 3 (Sirt3) maintains mitochondrial integrity and the expression of key FAO proteins (PPAR-alpha, CPT1A) via deacetylation of the LKB1-AMPKα axis; Sirt3 deficiency markedly worsens lipotoxicity, particularly through intracellular accumulation of free fatty acids such as palmitic acid. Piret et al. [40] identified Krüppel-like factor 15 (KLF15) as an essential transcriptional cofactor for PPAR-alpha: loss of KLF15 causes profound shutdown of metabolic pathways in proximal tubules and thereby exacerbates inflammation and fibrosis.

A opposing, pathological influence is exerted by fatty acid-binding proteins. FABP4, normally expressed at low levels in tubular epithelium, is dramatically upregulated after acute or nephrotoxic insults (Li et al. [38], Wang et al. [44]). Consistent data indicate that FABP4 acts as a proinflammatory amplifier: it suppresses protective PPAR-gamma expression and activates inflammatory cascades including NF-κB, STAT3, and the TLR4/MyD88/JNK pathway. Pharmacological inhibition of FABP4 with BMS309403, or genetic deletion of FABP4, significantly reduces inflammation, apoptosis, and fibrotic remodeling.

Finally, accumulation of specific lipid species matters. Xiong et al. [42] showed that downregulation of UCP1 leads to pathological triglyceride accumulation, which can be reversed by UCP1 agonism through AMPK/ULK1-induced autophagy. Deng et al. [47] implicated the fatty acid elongase Elovl6 in pathogenesis: Elovl6-mediated elongation of palmitic acid (C16:0) to stearic acid (C18:0) triggers severe ER stress with CHOP activation, underscoring that not only the amount but the precise molecular composition of the intracellular lipid pool determines cellular viability.

### Exogenous modulation of fatty acid metabolism

The therapeutic strategy of modifying the neuronal and renal lipidome by exogenous administration of specific fatty acids or their derivatives shows promise but yields highly context-dependent results. A classical approach is substituting omega-3 polyunsaturated fatty acids (PUFAs) (e.g., DHA or EPA) for omega-6 PUFAs (Hassan et al. [46], Tokumaru et al. [56]). Omega-3 fatty acids shift the balance away from proinflammatory eicosanoids toward protective lipid mediators (autacoids such as PD1, 17-HDHA, 18-HEPE, and 17,18-EpETE). These derivatives reduce polymorphonuclear neutrophil (PMN) infiltration, inhibit myofibroblast activation (reflected by reduced α-SMA), and induce cytoprotective proteins such as heme oxygenase-1 (HO-1). Newer formulation-based conjugates, for example the DHA-acylated curcumin ester Cur-2DHA described by Shi et al. [51], further demonstrate synergistic reductions in NLRP3 inflammasome activation and oxidative stress. However, the effects of PUFAs can be a metabolic tightrope, as shown by Shan et al. [52]. While a DHA-rich diet was protective in a rhabdomyolysis-induced AKI model, it dramatically worsened tissue injury in a folic-acid AKI model. Because PUFAs are highly susceptible to lipid peroxidation due to their multiple double bonds, they can under specific conditions (such as the folic-acid model) strongly promote ferroptosis. This finding underlines that exogenous lipid interventions must be carefully tailored to the underlying AKI etiology. Metabolically more stable alternatives include monounsaturated fatty acids (MUFAs) and specific lipid amides. Oleic acid (OA) produced marked antioxidant and antiapoptotic effects in an LPS model via Keap1/Nrf2 and Ras/MAPK/PPAR-γ signaling [50]. The endogenous lipid amide oleoylethanolamide (OEA) replicated these protective effects in the folic-acid model by stimulating PPAR-α–mediated peroxisomal activity and blocking epithelial-to-mesenchymal transition (EMT) via TGF-β1 [54]. Finally, the work by Deng et al. [47] on epoxide metabolites highlights the high complexity of closely related structural derivatives: the omega-6 arachidonic-acid derivative 14 [15]-EET exerted nephroprotection through stabilization of GSK3-β phosphorylation, whereas the structurally similar omega-3 DHA-derived 19 [20]-EDP aggravated morphological damage.

One additional limitation of the therapy studies should be briefly noted. In nearly all exogenous modulation studies (Hassan [46], Shi [51], Shan [52], Tokumaru [56], Silva [55]), diets or agents were given prophylactically - typically seven days before the insult. In clinical practice, however, acute kidney injury (AKI) usually occurs unpredictably. Whether these interventions are effective as rescue therapy after a manifest insult remains unresolved in most studies.

### Conclusions

In summary, restoring or metabolically bypassing renal fatty acid oxidation - whether by stimulating the peroxisomal axis, activating the key regulators Sirt3 and KLF15, blocking proinflammatory mediators such as FABP4, or precisely modulating dietary polyunsaturated and monounsaturated fatty acids (PUFAs/MUFAs) - represents a promising therapeutic approach to protect the renal proximal tubule. It needs nevertheless to be mentioned that optimizing lipid metabolism alone is unlikely to prevent progression to ATN unless perfusion and oxygen delivery are also restored, so reestablishing adequate blood flow remains the decisive therapy. When residual perfusion exists, improving lipid metabolism can most likely delay irreversible injury by reducing oxidative stress and stabilizing mitochondrial fatty-acid oxidation to maintain energy production and increase hypoxia tolerance. After reperfusion, optimized lipid handling may further support renal regeneration and lessen the development and severity of subsequent CKD. Before certain strategies can be tested clinically, they urgently require validation in therapeutic (post-injury) settings, precise determination of tissue concentrations, and the use of highly specific, biochemically optimized therapeutic compounds. Nevertheless, the presented data offers promising perspectives, particularly given the urgent need for more effective interventions for acute kidney injury (AKI). 

**Table 1:** Summary of all studies discussed in the text

Reference	Category	Design	Results	Limitations
Impairment of endogenous fatty acid metabolism				
Chiba et al., 2019 [32]	Endogenous impairment	Utilization of age-matched, male global Sirt5-deficient mice (Sirt5-/-) and littermate wild-type controls, ischemia-reperfusion injury (IRI) and cisplatin-induced toxicity; additional cell experiments on primary mouse proximal tubular epithelial cells (PTECs) and human proximal tubule cells (hPTECs) utilizing siRNA knockdown	Sirt5 was found to be highly enriched within proximal tubular epithelial cells. Following both ischemia- and cisplatin-induced AKI, mice lacking Sirt5 exhibited significantly preserved kidney function and markedly less structural tissue damage. Mechanistically, deleting Sirt5 moderately suppressed mitochondrial respiration but significantly enhanced peroxisomal fatty acid oxidation (FAO), thereby shifting metabolic reliance to the peroxisome to protect cells from injury.	Mice possessed a mixed C57BL/6 and 129 Sv genetic background; Long-term consequences and kidney fibrosis were assessed just 7 days post-injury.
Imafuku et al., 2020 [35]	Endogenous impairment	In vitro (HK-2 cells) and in vivo mouse study; cisplatin-AKI and 5/6 nephrectomy CKD model. Adenovirus-based Elovl6 overexpression; Elovl6 knockout model; ER-stress and AOPP analyses.	Renal stearic acid (C18:0) increased and palmitic acid (C16:0) decreased in AKI. Elovl6 activity rose concurrently. Elovl6 overexpression elevated C18:0 and cell damage. C18:0 more cytotoxic than C16:0; induced higher CHOP transcription (ER stress). Elovl6 knockout reduced kidney damage in CKD. AOPP implicated in Elovl6 activation. Stearic acid identified as a mediator of tubule-cell damage in AKI.	Use of immortalised HK-2 cells (may differ from primary human proximal tubule cells). Free fatty acids applied at 100–500 µM; whether such concentrations occur in vivo at interstitial/intracellular sites is unclear.
Li et al., 2020 [36]	Endogenous impairment	In vivo cisplatin-AKI mouse model (20 mg/kg IP, male mice) + cultured murine renal tubular epithelial cells. Sirt3 KO vs. WT; honokiol (Sirt3 agonist) pre-treatment. Biochemical, histological, metabolomic, and mitochondrial assays.	Cisplatin caused elevated creatinine/BUN, proximal tubular injury, lipid accumulation, increased FFAs (palmitic acid ~ 19×), ROS, lipid peroxidation, and reduced FAO proteins (PPARα, CPT1A, ACADL), TCA intermediates, and ATP. Sirt3 KO worsened all parameters. Honokiol restored Sirt3, FAO proteins, reduced FFA accumulation and tubular injury, improved ATP/mitochondrial morphology in WT but not Sirt3 KO. Mechanism: Sirt3 deacetylates LKB1 → LKB1–AMPKα activation → stimulated FAO.	(I) Palmitate used to model lipotoxicity in vitro, but in vivo fatty acids are protein-bound and present as complex mixtures, altering toxicity and cellular uptake. (II) Honokiol is a pleiotropic natural product with intrinsic antioxidant and cytoskeletal effects; off-target contributions in WT background cannot be fully excluded despite Sirt3-KO controls.
Li et al., 2021 [38]	Endogenous impairment	C57BL/6 and Fabp4-KO mice; five AKI/CKD models (folic acid, aristolochic acid, glycerol rhabdomyolysis, IRI, UUO). RNA-seq, pharmacologic inhibition (BMS309403, 40 mg/kg/day), biochemical and histological assays.	FABP4 expression markedly increased in injured kidneys (≈ 3× in some AKI models), localised to tubular epithelial cells. Pharmacologic inhibition or genetic deletion reduced serum creatinine/BUN, lessened tubular damage, decreased collagen deposition, and lowered proinflammatory cytokines (IL-6, MCP-1, TNF-α). Mechanism: restored PPARγ expression; suppressed NF-κB p65 and STAT3 phosphorylation.	Only 8-week-old male mice used (limited generalisability to females). Only early fibrosis assessed (48 h FA; day 5 AA); long-term CKD effects not evaluated. High-dose, abrupt toxin models (single 250 mg/kg FA; 5 mg/kg AA ×4 days) do not reflect gradual human kidney disease.
Piret et al., 2021 [40]	Endogenous impairment	Proximal-tubule–specific Klf15 KO mice (Pepck-Cre); AAI-AKI, IRI-uninephrectomy, and UUO models. RNA-seq, ChIP, primary PT cell metabolic assays, human CKD kidney samples.	PT KLF15 expression reduced after injury; its loss worsened creatinine/urea rise, PT dropout, inflammation, myofibroblast proliferation, collagen I/α-SMA deposition, and impaired recovery after IRI. RNA-seq: immune/integrin pathways upregulated; FAO, PPAR signalling, and amino-acid/carbohydrate metabolism downregulated. KLF15 and PPARα co-regulate FAO genes (CPT1A, ACAA2); KLF15 loss impaired palmitate-driven O₂ consumption. Human CKD data: reduced nuclear PT KLF15 correlated with eGFR independently.	(I) KLF15 overexpression alone did not fully restore FAO after AAI injury in vitro, indicating dependence on cofactors (e.g., PPARα), complicating therapeutic translation. (II) No in vivo evidence of therapeutic reversibility (e.g., inducible KLF15 overexpression or activators post-injury); rescue potential remains hypothetical.
Xiong et al., 2021 [42]	Endogenous impairment	Cisplatin-induced AKI mouse model. Lipid metabolomics, lentiviral UCP1 overexpression, UCP1 agonist CL316243. In vitro and in vivo assays (kidney morphology, function, inflammation, apoptosis, AMPK/ULK1/autophagy pathway).	AKI correlated with significant lipid (triglyceride) accumulation positively linked to injury severity. UCP1 significantly downregulated in AKI and negatively correlated with injury severity; expression localised to renal tubules. UCP1 overexpression (lentiviral or CL316243) reduced lipid accumulation, alleviated injury markers, inflammation, and apoptosis. Mechanism: UCP1 upregulation activated AMPK/ULK1/autophagy pathway, improving cellular function.	CL316243 is a β3-adrenergic receptor agonist; systemic administration can cause widespread metabolic and cardiovascular effects (e.g., systemic lipolysis in adipose tissue) that may indirectly influence the kidney, making renal-specific attribution difficult.
Wang et al., 2022 [44]	Endogenous impairment	CLP (cecal ligation and puncture) septic AKI mouse model. Fabp4-KO mice and BMS309403 pharmacologic inhibition. Renal-tubule–specific Fabp4 deletion. In vitro TCMK-1 cells with LPS stimulation.	FABP4 elevated in CLP kidneys, correlating with inflammation and apoptosis. Fabp4-KO or BMS309403 improved kidney function and reduced injury, inflammation, and apoptosis. FABP4 upregulation linked to TLR4/MyD88/JNK/c-Jun signalling. Tubule-specific Fabp4 deletion mitigated CLP-induced AKI. Positive feedback loop between FABP4 and c-Jun identified.	BMS309403 may exhibit off-target effects (minor cross-reactivity with FABP3, FABP5); complete in vivo specificity of the compound cannot be guaranteed.
Chiba et al., 2024 [33]	Endogenous impairment	In vivo mouse study; LCAD-/- mice vs. WT in cisplatin-induced nephrotoxicity and IRI models. Biochemical, histological, mitochondrial-respiration, and lipidomic assays.	LCAD-/- mice were significantly protected against both AKI models: preserved renal function, less tubular damage, reduced lipid peroxidation, lower ROS, decreased cell death, attenuated ferroptosis pathways, and maintained mitochondrial respiratory capacity. Proposed compensatory upregulation of peroxisomal FAO (PPARα-like) as protective mechanism.	Peroxisomal FAO inferred indirectly (etomoxir, off-target effects); constitutive - not pharmacologic - LCAD knockout; Sirt5–LCAD axis not directly examined; IRI used only males; ferroptosis assessed with non-specific TUNEL + few mRNA markers; small n, mixed genetic background; only short-term outcomes reported.
Exogenous modulation of fatty acid metabolism				
Hassan et al., 2009 [46]	Exogenous modulation	Murine renal IRI model; menhaden oil (omega-3 PUFA) vs. corn oil (omega-6 PUFA) diet. Assessment of renal function, PMN infiltration, cytokines/chemokines, eicosanoids, and DHA-derived autacoids (PD1, 17-HDHA). In vitro mesangial cell HO-1 assays.	Omega-3 PUFA diet improved kidney function and survival after ischaemia; reduced PMN infiltration, inflammatory cytokines/chemokines, and harmful eicosanoids; increased protective DHA-derived autacoids (PD1, 17-HDHA) and renal HO-1 expression. Omega-6 diet caused high mortality and increased inflammation. Autacoids alone (systemic) reduced PMN recruitment and enhanced HO-1 in vivo and in vitro.	Tissue/blood collected only at 24 h post-ischaemia; no data on longer-term survival, chronic structural remodelling, or fibrosis. 100% mortality in omega-6 group at 45 min ischaemia precluded cross-group comparison of repair mechanisms over extended timeline.
Deng et al., 2017 [47]	Exogenous modulation	Murine renal IRI model. Two FA metabolites compared: 19(20)-EDP (DHA epoxide, omega-3) vs. 14(15)-EET (ARA epoxide, omega-6). In vivo functional assessment (BUN, creatinine, NGAL); in vitro mRTEC hypoxia model (GSK3β phosphorylation, apoptosis).	14(15)-EET was nephroprotective (improved renal function, reduced apoptosis, preserved GSK3β phosphorylation in vitro). 19(20)-EDP exacerbated initial kidney injury. Effects reproduced in vitro. Study identified 14(15)-EET as a novel nephroprotective FA metabolite and demonstrated opposing effects of structurally related omega-3 and omega-6 epoxides.	In vitro, 19(20)-EDP dose-dependently reduced GSK3β phosphorylation in mRTECs; however, in vivo, 19(20)-EDP alone failed to significantly alter kidney-tissue GSK3β phosphorylation, indicating a translational gap between cell culture and whole-organ findings.
Zhang et al., 2022 [50]	Exogenous modulation	LPS-induced AKI mouse model. Oleic acid (OA) treatment in vivo and in NRK-52e cells. Metabolomic analysis (serum biomarkers); pathway analyses (Keap1/Nrf2, Ras/MAPK/PPARγ). Ras inhibitor FTS used to confirm signalling.	OA improved renal histopathology, lowered creatinine/urea, normalised immune-cell proportions, reduced pro-inflammatory cytokines (IL-2, TNF, IFN-γ), increased IL-10, decreased iNOS/COX-2/NF-κB p-p65. Reduced ROS, inhibited apoptosis (↓caspase-9/3, ↓Bax, ↑Bcl-2). In cells: ↓ROS, ↑mitochondrial membrane potential, ↑Ras/Raf1/SHC/PPARγ. Metabolomics identified 30 differential biomarkers and 10 altered pathways.	No validated positive control/reference drug for AKI used for comparison. Western blot for key apoptotic, MAPK, and PPARγ components limited to *n* = 3 mice per group, severely limiting statistical power and generalisability of pathway findings.
Shi et al., 2022 [51]	Exogenous modulation	Cisplatin-induced AKI mouse model. Dietary interventions: Cur-2DHA (DHA-acylated curcumin diester), Cur-DHA (monoester), and controls. 7-day pre-treatment before cisplatin. Assessment of kidney function, histopathology, oxidative stress (GSH, MDA), NLRP3 inflammasome pathway, and renal fatty acid composition.	Cisplatin caused weight loss, reduced food intake, elevated BUN (2.31×) and Cr (1.03×), severe tubular necrosis/inflammation. Cur-2DHA significantly reduced BUN (71.7%) and Cr (48.1%), alleviated histopathology, improved GSH, reduced MDA. Cur-2DHA inhibited TNF-α, IL-1β, IL-6, and NLRP3-related proteins. Improved renal fatty acid profile (specific FA ratios stabilised).	Mice were pre-treated for 7 days before cisplatin. In clinical practice, AKI is mostly unpredictable or diagnosed after injury onset. The study therefore demonstrates a prophylactic rather than a therapeutic (post-injury) effect.
Shan et al., 2023 [52]	Exogenous modulation	Two AKI models (rhabdomyolysis [Rha-AKI] and folic acid [FA-AKI]) in mice. 7-day dietary supplements: DHA-rich fish oil, oleic-acid–rich camellia seed oil, or lard (SFA). Ferrostatin-1 (Fer-1) ferroptosis-inhibitor control. In vitro HK-2 cells (RSL3/erastin ferroptosis; myoglobin Fe²⁺ cytotoxicity).	Fer-1 protected against FA-AKI but not Rha-AKI (ferroptosis involved in FA-AKI only). DHA accumulated in kidney. In Rha-AKI: DHA improved kidney function, reduced histologic injury, lipid-peroxidation markers (MDA, 4-HNE), and inflammation; mitigated hyperlipidaemia. In FA-AKI: DHA worsened injury (greater tubular loss, higher BUN/creatinine, more MDA/4-HNE, more fibrosis, poorer recovery). In vitro: DHA did not affect myoglobin cytotoxicity but exacerbated RSL3/erastin ferroptosis (reversed by Fer-1). Context-dependent DHA effects demonstrated.	Clinical AKI rarely has a single, clear-cut aetiology; real patients frequently present with overlapping insults where multiple cell death pathways (apoptosis, necroptosis, ferroptosis) coexist simultaneously, limiting direct translational inference.
Comella et al., 2024 [54]	Exogenous modulation	Folic-acid–induced AKI mouse model. OEA (oleoylethanolamide) treatment. PPARα-KO mice to confirm mechanistic pathway. In vitro HK-2 cells with TGF-β1 stimulation. Outcomes: renal function, histology, inflammation, fibrosis, EMT markers.	OEA normalised FA-induced kidney dysfunction (reduced creatinine, urea, albumin excretion). Alleviated tubular damage, reduced inflammation markers and tubular injury markers, curtailed renal fibrosis (↓fibrosis genes, ↓collagen deposition). Mechanism: PPARα activation regulating peroxisomal β-oxidation confirmed by absent protection in PPARα-KO mice. In HK-2 cells: OEA inhibited TGF-β1–induced EMT and fibrosis markers via PPARα-dependent pathway.	Despite PPARα-KO mice showing absent OEA benefit, a non-significant trend toward reduced inflammatory/fibrotic markers remained. Alternative OEA receptors (TRPV1, GPR119) may contribute to protective effects but were not experimentally isolated or controlled.
Silva et al., 2024 [55]	Exogenous modulation	Wild-type male mice; 7-day dietary DC8 supplementation (5% or 10% w/w) before IRI (unilateral + delayed contralateral nephrectomy) or cisplatin AKI. Global DIA-MS proteomics (~ 4,000 proteins, > 4,500 succinylated peptides), radiolabelled FAO assays, immunofluorescence, mitochondrial-respiration assays.	DC8 induced marked lysine succinylation specifically in kidney (peaked ~ 72 h, washed out by day 5). DC8 strongly protected against both IRI and cisplatin AKI: lower creatinine/BUN, reduced NGAL, preserved tubular morphology, fewer TUNEL+ cells. Mechanisms: blunted mitochondrial protein loss, preserved complex I subunits and complex I–driven respiration; massively increased peroxisomal protein succinylation and abundance, particularly FAO enzymes; preserved peroxisomes after IRI. Functional assays confirmed increased peroxisomal FAO.	(I) DC8 was administered 7 days pre-AKI; clinical scenarios typically present post-insult, so whether DC8 works as rescue therapy post-injury is unknown. (II) Effective dose was 5–10% w/w of diet as mush; translating this mass requirement to humans poses severe logistical and palatability challenges for clinical trials.
Tokumaru et al., 2024 [56]	Exogenous modulation	Mice fed linseed oil (high omega-3) or soybean oil (low omega-3) for 4 weeks before renal IRI. UUO model for anti-fibrotic confirmation. Metabolomic analysis (LC-MS). In vitro: cultured rat renal fibroblasts treated with EPA metabolites 18-HEPE and 17,18-EpETE.	Linseed oil diet raised renal EPA levels, improved post-IRI survival, reduced BUN/creatinine, lessened tubular injury and fibrosis. Anti-fibrotic effects confirmed in UUO model (reduced fibrosis and myofibroblast markers). Metabolomics: ↑EPA metabolites (18-HEPE, 17,18-EpETE), ↓AA metabolites. 18-HEPE and 17,18-EpETE inhibited α-SMA expression in renal fibroblasts, suggesting a mechanistic pathway.	LC-MS analysis failed to detect resolvins (including Resolvin E1, the downstream product of 18-HEPE) in renal tissue, leaving the precise downstream metabolic pathway partially unverified due to methodological limitations.

## Data Availability

No datasets were generated or analysed during the current study.
